# Applicability Domain of the Sens-Is In Vitro Assay for Testing the Skin Sensitization Potential of Rheology-Modifying Polymers

**DOI:** 10.3390/polym17101408

**Published:** 2025-05-20

**Authors:** Isabelle Hochar, Mickaël Puginier, Hervé Groux, Jérôme Guilbot, Françoise Cottrez, Alicia Roso

**Affiliations:** 1 Seppic Regulatory, 50 Boulevard National, 92257 La Garenne Colombes, France; isabelle.hochar@airliquide.com (I.H.); mickael.puginier@airliquide.com (M.P.); 2 IMMUNOSEARCH, Les Cyclades, Chemin de Camperousse, 06130 Grasse, France; hgroux@immunosearch.fr (H.G.); fcottrez@immunosearch.fr (F.C.); 3 Seppic Research & Innovation, 127 Chemin de la Poudrerie, 81105 Castres, France

**Keywords:** rheology-modifying polymers, skin sensitization, polysaccharides, synthetic polymers, plant-based polymer, Sens-Is assay, cosmetics

## Abstract

Assessing the propensity of ingredients to induce skin sensitization through in vitro testing is crucial for worker and consumer safety. This is particularly important for novel and high-performance ingredients with complex structures, such as rheology-modifying polymers, which are extensively used in cosmetics, pharmaceuticals, and detergents. The Sens-Is assay has proven effective in distinguishing skin sensitizers from non-sensitizers for difficult-to-test ingredients when integrated into a multi-method in vitro approach. Therefore, the primary goal of this research was to explore whether the Sens-Is in vitro assay is suitable to evaluate rheology-modifying polymers. Fifteen structurally diverse rheology-modifying polymers, including natural polymers obtained by extraction, chemical synthesis, or biotechnology, spanning varying physical forms and concentrations, were evaluated. The results showed that most polymers were non-sensitizing, consistent with available in vivo data. Although polymer macromolecules generally exhibit limited skin sensitization potential due to their surface confinement, the Sens-Is assay permitted the detection of weak signals from secondary components or possible byproducts in specific cases. This work confirms Sens-Is as a useful tool in an overall approach to assessing the skin sensitization liability of polymers under development, but careful solvent selection is crucial to ensure accurate results and prevent potential overexposure due to polymer retention on the epidermal surface.

## 1. Introduction

Allergic Contact Dermatitis (ACD) is an inflammatory skin condition caused by a type IV hypersensitivity reaction. This reaction results from an individual’s immune system response to a low molecular weight compound (less than 500 Daltons (Da)) or hapten, leading to cutaneous manifestations such as rash, erythema, eczema, or vesicular dermatitis [1].

Epidemiologically, ACD represents a leading form of work-related dermatological disorder [2]. Data indicate a growing incidence of ACD across global regions, particularly within Europe. An analysis of 28 distinct investigations, spanning a decade from 2007 to 2017 and encompassing over 20,000 individuals undergoing patch testing, revealed an approximate overall prevalence of 20% within European cohorts [3]. Subgroup analysis demonstrated that around 16% of pediatric and adolescent populations, 27.9% of women, and 13% of men were affected, reflecting that the condition is more common in women than in men. In the elderly, the condition is often associated with topical medications.

Over the past five decades, the dermatology community has observed a distinct evolution in the nature of allergens, including the frequent and rapid identification of previously unknown sensitizing substances [4].

This necessitates the assessment of skin sensitization potential for all topical ingredients. With the implementation of the European Cosmetics Regulation (EC) no 1223/2009, which enforced the prohibition on animal testing in the cosmetics sector, alternative in vitro tests have been established in recent years. Skin sensitization is a complex physiological phenomenon described as an Adverse Outcome Pathway (AOP) developed and published by the Organization for Economic Co-operation and Development (OECD) in 2012 [5,6]. Within the framework of the AOP, a cascade of sequentially dependent Key Events (KEs) occurs across various biological tiers, ultimately leading to an adverse health outcome. For this specific toxicological endpoint, four events are recognized as key, subsequent to the prerequisite of skin permeation: (1) haptens binding to skin proteins; (2) keratinocyte stimulation leading to increased levels of inflammatory mediators and activation of cellular defense mechanisms; (3) dendritic cell elicitation associated with the expression of specific cell surface markers, chemokines, and cytokines; and (4) the final stage of memory T cell stimulation and multiplication. In order to investigate critical key events one to three within the skin sensitization pathway, a series of in chemico and in vitro assays have been established and some of them have been validated as OECD Test Guidelines. These methods, known as New Approach Methodologies (NAMs), offer mechanistic insights into skin sensitization, thereby supporting the transition from traditional animal testing [7]. Since there is currently no single test that can be used as a stand-alone alternative to in vivo testing [8], a combination of multiple tests covering critical KE of the AOP is required. The OECD 497 regulatory guideline, updated in July 2023, presents Defined Approaches with potential combinations of NAMs to conclude on skin sensitization hazard. The guideline describes two main Defined Approaches: the ‘2-out-of-3 approach’ and the ‘Integrated Testing Strategy’. However, both these strategies are primarily applicable to easy-to-test compounds, such as mono-constituent ingredients.

To ensure the safety of challenging ingredients, ingredients with a water solubility of less than 60 mg/L, surface-active agents, and/or complex substances (Unknown or Variable composition, Complex reaction products, or Biological materials called UVCB) [8], we have developed a specific three-step strategy using ‘2-out-of-3 approach’. This strategy is based on the Sens-Is assay as a starting point, covering key events 1 and 2, followed by the h-CLAT model, covering key event 3. In case of conflicting results, a third test is performed (KeratinoSens). The underlying concept of Sens-Is is built upon research showing that sensitizing chemicals induce specific gene expression patterns in skin cells, particularly in keratinocytes [9]. The method’s description and proof of concept were developed in the mid-2010s [10]. Large-scale formal validation began in 2016 [11], and studies later demonstrated the method’s ability to accurately predict sensitization potency [12,13]. The applicability domain of the Sens-Is has been investigated across a diverse range of chemical classes and functionalities [14]. These tests included fragrance substances (e.g., L-Limonene, Citronellol), preservatives (e.g., parabens), active ingredients derived from plant and algal extracts, emollients (e.g., Isopropyl myristate), and both ionic and non-ionic surfactants (e.g., Cocamidopropyl betaine, Stearylglyceryl sugar lipid) [15,16,17,18]. Polymeric structures were included in the validation and further investigations: certain polymeric surfactants, such as Polysorbates [11,16], and polymeric silicones, including amine-functionalized structures, used as emollients or hair conditioners, were subjected to specific experiments, which demonstrated satisfactory predictive capacity [19]. Additional investigations were conducted on ethoxylated fatty alcohols, one polyurethane, polyquaternium-derived polymers, and acrylic and vinylic copolymers with varying molecular weights [18]. The investigations revealed a technical limitation for one cationic polymer, which exhibited binding to genetic material. Furthermore, there were 4 out of 14 instances where the predictions did not align with in vivo results, including two false positives for polyquaterniums and one for a high molecular weight acrylate copolymer. Upon closer examination, it was noted that two of the tested polymers could potentially belong to the rheology modifiers category. Therefore, available in vitro skin sensitization assessment for rheology modifiers appeared limited, and investigation of natural thickeners and rheology modifiers seemed lacking. Previous studies have also shown the ability of the Sens-Is method to predict the effects of mixtures (i.e., combinations of non-sensitizers could induce a sensitization response) and to assess formulated finished products, particularly cosmetics [15]. However, these studies highlighted the influence of formulation vehicles on the sensitization potential of known allergens. The method was further demonstrated to be capable of detecting allergens incorporated into silicone devices following an appropriate extraction procedure [20].

Polymers, characterized by repeated structural units (i.e., monomers), are part of difficult-to-test ingredients. Such compounds exhibit a distribution of molecular weight values [21]. It is assumed that their high molecular weights (>1000 Da) prevent these substances from penetrating the skin barrier. Nevertheless, low molecular oligomers and potential conformation changes, impurities, and residues may have a greater potential for absorption and penetration into the skin [22], which may trigger a skin allergic reaction. In cosmetic formulations, the structural diversity of polymers provides a range of functionalities such as rheology and viscosity control, foam regulation (enhancing stability or promoting collapse), emulsion stabilization, hair styling benefits, film formation, and conditioning benefits [23], explaining their widespread and essential use in personal care products. Polymers can also have different origins, as they can be extracted from plants, made by synthesis, or derived from biotechnology, which adds an additional level of complexity to this particular type of substance. In the field of rheology-modifiers dedicated to topically applied formulations, synthetic polymers are renowned for their versatility and stabilizing performance [24,25]. Their ability to stabilize oils makes it easy to design cold-manufactured cream-gel formulations, without the need for additional surfactant [26,27]. Natural rheology-modifiers are also widely used [28] with renewed interest in recent years driven by consumer demand for more natural products. However, the performance of these natural polymers is less versatile, being used mostly to thicken and co-stabilize formulations in combination with surfactants (i.e., emulsion form). The market context and technical concerns for the formulators encourage active searching for solutions that have a high natural content [29]. There are several ways to approach this research, including the pursuit of new polymeric structures that come from nature and have better performance, and the development of synthetic-natural hybrid polymers [30,31]. Regardless of the research direction and beyond the complex composition of plant-based polymers, the addition of natural resources can lead to the presence of unpredictable impurities, which may be subject to variations depending on the climate or the area where the plant was collected [32,33,34,35]. Therefore, there is a real need to detect potential skin sensitization in these new structures, although it is challenging to assess the predictability of in vitro tests.

Given Sens-Is’ ability to take into account the reality of the final application on skin and considering the limited data on existing rheology-modifying polymers, this work aimed to determine the applicability of this method for assessing the potential sensitizing effects of rheology-modifying polymers. Additionally, this research aimed to evaluate the capacity of the Sens-Is assay to detect sensitizing impurities or secondary components in the polymers. Ultimately, considering ongoing research into hybrid synthetic-natural structures, mixtures, or chemically or biotechnologically modified natural polymers, the purpose was to determine whether incorporating this test into a multi-test strategy would help prevent potential skin sensitization issues in the development of new rheology-modifying polymers for topical products.

## 2. Materials and Methods

### 2.1. Materials

A set of fifteen anionic or non-ionic polymers designed to modify rheology or to provide a texturing effect (six synthetic in origin and ten of natural origin) was tested according to the Sens-Is protocol (Table 1). Synthetic polymers (C1 to C5), polysaccharides (N1 and N5), and a plant-based hydrophobic polymer (N6) were sourced from Seppic (Castres, France). Polysaccharide N2’ was sourced from Tate & Lyle (London, UK). Due to ethical considerations concerning the findings, the supplier of polysaccharide N2 remains confidential. Polysaccharide N3 was obtained from Atina (Sao Paulo, Brazil), and N4 from Nagase GmbH (Düsseldorf, Germany). Polymers R1, R2, and R3 were included as reference materials (non-sensitizers R1 and R3 were obtained from Merck KGaA (Darmstadt, Germany). R2 was sourced from Alban Müller (Chartres, France). Data indicated that R1 was not sensitizing to guinea-pigs’ skin [36]. Based on its established toxicological profile [37], R2 was presumed to be non-sensitizing. The non-sensitizing potential of R3 was presumed through read-across from linear polysaccharides and their salts [38]. Moreover, they are widely used in cosmetic products [39,40,41] with limited cosmetovigilance data published, implying a low sensitization risk under normal and intended conditions of use.

These references were not expected to be sensitizers [25,26,27]. As high molecular weight rheology-modifiers are unlikely to permeate the skin and induce skin sensitization, a monomer, 2-Ethylhexyl acrylate, characterized as a moderate skin sensitizer in vivo [42], was included as a positive reference (PR; Merck KGaA, Darmstadt, Germany). Residual monomer traces (such as this substance) could be found as a typical impurity when the polymerization process is still incomplete.

**Figure 1:**
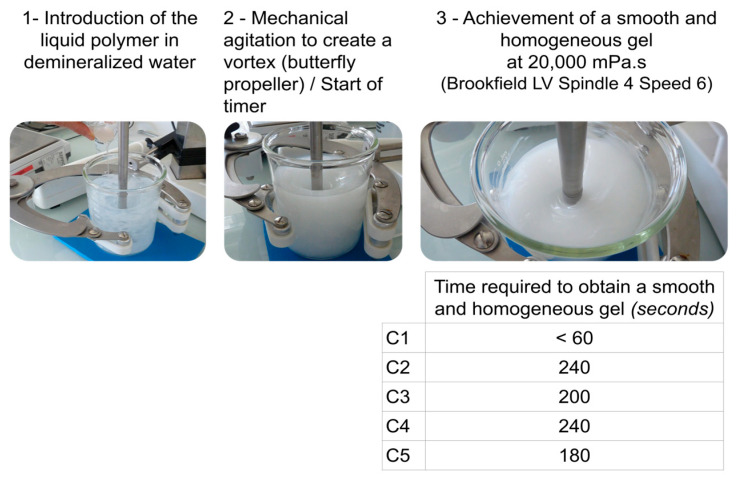
Manufacturing of Aqueous Gels from inverse emulsion polymers: protocol illustration and time required to obtain a smooth hydrogel in seconds. Gel homogeneity was determined through visual inspection for the absence of visible aggregates or lumps. Internal consistency was verified by spatula extraction. Agitation continued until no particulates were detected.

A variety of polymers were selected to be as representative as possible of the broad range of rheology-modifiers available of different chemical natures; obtained by different polymerization technologies for the synthetics (Cn; polymerization in inverse emulsion or precipitation polymerization); from different plant resources and extracted from different parts of the plant (trunk exudate, seed, or root) for the naturals (N1, N3, N4, N5, R1, R3); and obtained by biotechnology (N2, N2’, and R2). These polymers were supplied either in liquid form (in various concentrations) or in pure powder form. The Convention on Biodiversity provided the framework for our natural polymer research initiatives. The natural origin tested items were sourced and used in full conformity with the Nagoya Protocol and specific national laws relating to access to genetic resources and the sharing of benefits arising from their use. The status of plant and marine resources was continuously monitored with respect to the Convention on International Trade in Endangered Species of Wild Fauna and Flora and the International Union for Conservation of Nature. Although less concentrated materials are available, polymerization in inverse emulsion (i.e., water-in-oil) provides pre-neutralized ready-to-use materials [43]. The radical polymerization process is carried out in the emulsion, without solvent. Interestingly, it does not require high-energy consumption and does not generate waste. The emulsion excipient contains a surfactant (called an inverter), which reverses the emulsion when water is added. The polymer is then released into the water phase and swells under mixing in less than five minutes to create smooth and homogeneous hydrogels, (Figure 1), instead of the ten to twenty minutes required for powder forms [44]. For these synthetic polymers, the emulsion components of fossil origin present in the historical variant (Control C2) are replaced by suitable plant-based raw materials (C1, C3, C4, and C5) to increase the ‘natural’ content while maintaining similar ease of use and application performance [45].

### 2.2. Sens-Is Assay

**Figure 2:**
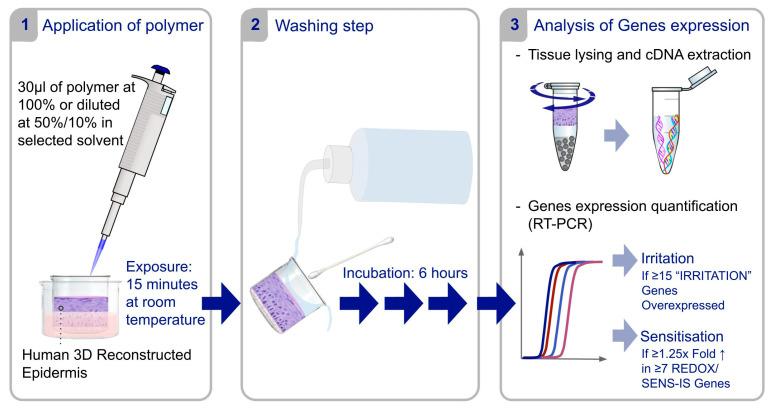
Summary of the Sens-Is assay protocol.

The Sens-Is assay (Figure 2) is an in vitro method designed to analyze the expression of specific genes associated with biological mechanisms of skin sensitization [10] in a reconstructed human epidermis model (Episkin^®^, Lyon, France) exposed to various doses of test chemicals. This assay monitors two sets of genes involved in skin sensitization mechanisms: the ‘REDOX’ group, focused on the Keap1-Nrf2-ARE dependent pathway, activated by chemicals binding to cysteine residues, and the ‘SENS-IS’ group, involving pathways that cause dendritic cell activation through exclusive chemical attachment to lysine residues. Notably, a third set of genes within the Sens-Is assay allows for the differentiation of irritant responses from sensitizing reactions. Skin irritation is a direct and reversible response caused by local damage. In contrast, skin sensitization is an irreversible immune-mediated reaction triggered by repeated allergen contact, highlighting the importance of identifying potential sensitizers [46]. The testing was conducted according to the ImmunoSearch Standard Operating Protocol (SOP). To summarize, test items (30 μL) were applied to tissues for 15 min. The chemical was used undiluted, if feasible, or diluted at 50% and 10% in Phosphate Buffered Saline (PBS, PAN-Biotech GmbH, Aidenbach, Germany), olive oil (Puget, Paris, France), DiMethyl SulfOxide (DMSO, PAN-Biotech GmbH, Aidenbach, Germany), or Dipropylene Glycol (DPG, Merck KGaA, Darmstadt, Germany). The solvent was chosen for its ability to create a homogeneous preparation, free of precipitate, based on visual inspection. After washing and 6-h post-incubation, tissues are processed for complementary Deoxyribonucleic Acid (cDNA) quantification using Reverse Transcriptase—Polymerase Chain Reaction (RT-PCR).

Each investigation included three negative controls (PBS, olive oil, and DMSO), a positive irritation control (Sodium Lauryl Sulfate, CAS No. 151-21-3, Merck KGaA, Darmstadt, Germany) at 5%, and a positive sensitization control (2,4,6-trinitrobenzene sulfonic acid, TNBS; CAS No. 2508-19-2, Merck KGaA, Darmstadt, Germany) at 1%. A test item was defined as sensitizing when the data revealed that seven or more genes from the REDOX or SENS-IS collections exhibited a minimum 1.25-fold increase in expression over the control samples. A test item was defined as irritating if it induced the overexpression of at least 15 genes within the “IRRITATION” gene group. In this latter case, the test should be repeated at a lower concentration. In addition, a potency prediction can be made by identifying the lowest concentration that causes gene overexpression (strong, weak, or very weak sensitizer, when the test item is tested at 10%, 50%, and 100%, respectively).

## 3. Results

The test conditions (solvents employed and test item concentrations) and the resulting data for the rheology modifiers and the monomer detailed in Table 1 are summarized in Table 2.

The 15 polymers and the monomer were tested undiluted and/or at dilutions of 50% and/or 10%, according to the standard operating procedure (SOP). Solvents were chosen based on their capacity to optimize the application of each polymer to the reconstructed epidermis. Solvent selection was essential, as it could significantly influence the physical state of the polymer at the epidermal surface (visually observed effects). At the 50% concentration, three different solvents were used to test C1, with the aim of identifying the solvent that would most effectively facilitate polymer application: DPG, DMSO, and PBS. DMSO was found to be the solvent of choice and was subsequently used for preparing the majority of the rheology-modifiers, followed by PBS and finally DPG and olive oil.

As expected, the three natural polymers chosen as references (R1, R2, and R3) were non-sensitizing when tested at 100% in two different solvents (DMSO and PBS).

The positive reference (PR) was concluded to be a moderate sensitizer as it was positive at 50% and 10% in olive oil and DPG. Thirteen polymers did not show any potential sensitizing effect.

Among the synthetic polymers (C1 to C5) obtained by the same process of polymerization in inverse emulsion, only C3 proved to be a very weak sensitizer as it was tested positive at 100% in DMSO, whereas its homolog, C4, was non-sensitizing under similar conditions and in another solvent (i.e., in PBS).

Among the polysaccharides and plant-based polymers (N1 to N6), only N2 appeared to be a very weak sensitizer as it was found to be positive at 100% in DMSO, unlike N2’, which has the same INCI name but came from a different source.

## 4. Discussion

This work focused on analyzing the response of the Sens-Is assay to rheology-modifiers and its applicability for these difficult-to-test substances. To this end, the available in vivo data (OECD 406 (historical data), OECD 429 (historical data), HRIPT: Human Repeated Insult Patch Test) on these structures have been reported in Table 3 to determine whether there is a correlation with the Sens-Is results. Prior to conducting clinical trials, a safety assessment confirmed the limited skin sensitization potential in volunteers.

In vivo tests are available for most polymer materials. The results shown in Table 2 and Table 3 indicate that the conclusions of the Sens-Is tests are consistent with those obtained in vivo, except in two cases where the conclusions differed between the in vitro and in vivo results.

The Sens-Is assay correctly matched the in vivo potency of the PR, confirming the good sensitivity of the assay. The results for the three references R1, R2, and R3 agreed with the expected historical [39,40,41] and in vivo data (OECD 406) (Table 3).

The Sens-Is results for the synthetic rheology-modifiers aligned with the skin sensitization conclusions, with the exception of C3.

Experimental polymer C3 was shown to be a very weak sensitizer in the Sens-Is assay when diluted in DMSO (Table 2). This result was not in line with expectations when comparing this in vitro result with those obtained in vivo on a similar polymer by read-across [54]. Investigations were therefore carried out, indicating that this result was related to the inverter. A Sens-Is test performed on I2, the inverter of C3, resulted in a weak sensitizer classification (positive at 100% and 50% in DMSO), supporting the hypothesis. This hypothesis was prompted by a reported case of Allergic Contact Dermatitis to Polyglyceryl-10 laurate present in a gel [56]. Another source of Polyglyceryl-10 laurate (I1) was then tested, based on the assumption that subtleties in the manufacturing process or an impurity might be involved. The Sens-Is assay performed on I1 showed no sensitization when tested pure and diluted at 50% and 1% in PBS. This result highlights the variability between two sources of polyglyceryl-10 laurate with the same INCI name and chemical structure but different sources and manufacturing processes. These variations may be a result of either the polymerization of glycerol or the subsequent esterification process. Although checks showed that the numbers of ester bonds were very similar for I1 and I2, the distribution of the esters obtained could have been different.

Following these results, an optimized polymer was developed (C4), keeping the same polymeric structure but using another inverter. This polymer features 60% natural content according to the ISO 16128 standard [57,58] and has reduced environmental impact due to locally sourced oils, which lowered transport-related carbon emissions compared to the initial petroleum-sourced polymer. A Sens-Is assay performed on its inverter, Polyglyceryl-6 Laurate (less hydrophilic), showed no sensitization when tested pure and diluted at 50% in PBS, DMSO, and DPG, and a Sens-Is assay performed on C4 led to the same conclusion. These results agreed with the skin sensitization assessment (Table 3).

Of the 10 polysaccharides and plant-based hydrophobic polymers tested, the Sens-Is results were consistent with the skin sensitization conclusions, with N2 being the exception, demonstrating the general suitability of the assay for assessing natural polymers.

N2, tested pure and diluted in DMSO and PBS, was concluded to be a very weak sensitizer, whereas N2’, from another source, tested pure and diluted in DMSO, was concluded to be a non-sensitizer. These results contradict those found in the literature stating that the polymer is non-sensitizing [51]. Xanthan gum is an extracellular heteropolysaccharide synthesized by *Xanthomonas* species during aerobic fermentation. Multiple parameters significantly impact the industrial-scale biosynthesis of xanthan gum by microorganisms. Such parameters encompass the selected strain, nutrient availability (carbon and nitrogen), fermentation mode (batch or fed-batch), acidity, thermal conditions, initial microbial population density, oxygen supply, stirring, and the duration of culture growth [59]. All these variables may trigger differences in the rheological profile and quality of xanthan gum from one variant to another.

**Figure 3:**
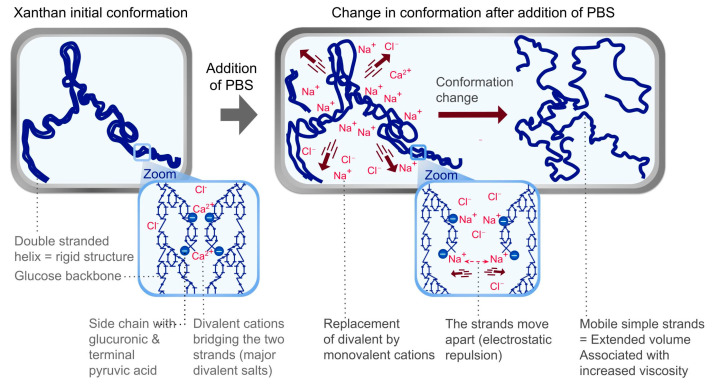
Influence of salts on xanthan gum conformation: a hypothesis explaining the increased viscosity and rinsing difficulty of preparations with polymer N2 during the Sens-Is assay.

The structure of this biopolymer is complex, with a D-Glucose backbone and a side chain of D-mannose, D-glucuronic acid, and D-mannose units. In addition, acetyl groups are substituents on the internal mannose units, while pyruvic acid moieties are attached to terminal mannose units [60]. Several hypotheses were considered. Although the Sens-Is assay does not target protein-related mechanisms, the nitrogen content was analyzed to estimate protein levels in N2 and N2’. Since these levels were similar, the hypothesis was not pursued further. Another hypothesis considered was the presence of traces of free pyruvate as the sodium salt, which may be associated with a sensitizing effect, the latter being classified as a cat.1B skin sensitizer (ECHA). Pyruvic acid moieties are connected to D-mannose as an acetal (i.e., covalent bond) [60]. This bond is not expected to be affected by Sens-Is testing conditions. A buffered medium and a pH indicator ensured that no changes occurred during handling, thus preventing any risk of hydrolysis [61]. In addition, the detection of free pyruvate was attempted in both samples without observing the characteristic signal (NMR technique: Nuclear Magnetic Resonance). Further analytical investigations on the composition will need to be conducted to determine whether variations in the manufacturing process and composition may actually affect the test result. A further hypothesis was prompted by the adhesion properties of this gum, in particular to biological membranes [62,63,64], that could affect rinsing of the epidermis, ultimately resulting in prolonged contact and overestimated results. Based on published X-ray diffraction analyses, xanthan gum conformation in powders may vary from amorphous to crystalline, depending on sources, which may influence the response [65]. Moreover, under diluted conditions, variations were observed in the thickening effect depending on the manufacturing process. This observation was consistent with previous studies and explained by a variable ratio between pyruvate and acetyl groups in the side chains (i.e., higher pyruvate content in the structure correlates with increased viscosity) [66,67]. In our investigation, a 1% aqueous solution at pH 6 of N2 showed a notably higher viscosity (5000 mPa·s) than that of N2’ (2900 mPa·s) when tested using a Brookfield LV Viscometer with Spindle 2 at Speed 6 (Appendix A, Table A1). These results suggested potential differences between the two polysaccharides, though they were not directly linked to the testing. Regardless of the adhesion properties of the gum, variations in viscosity could result in more or less effective rinsing. Furthermore, rinsing challenges may arise from the presence of salts in the Phosphate Buffered Saline (PBS), which was visually observed to increase viscosity. In the absence of additional measurements and without knowing the manufacturing process of the gums, the reported variability in xanthan conformations across different suppliers makes it difficult to formulate explanatory hypotheses. These conformations include a single-stranded form, predominantly associated with monovalent counter-ions, and a double-helical form, primarily associated with divalent cations. The single-stranded form, being more mobile, typically results in solutions with high viscosity, whereas the more compact double-helical form exhibits lesser thickening properties [68]. However, introducing salts into the surrounding environment has the potential to reverse spatial organization. PBS, which closely approximates the physiological ion concentrations of the human body, contains predominantly monovalent salts (i.e., sodium chloride 0.137 M, potassium chloride 0.0027 M, disodium phosphate 0.01 M, and potassium dihydrogen phosphate 0.0018 M). This composition may have induced a transition from the initial double-stranded to the single-stranded conformation, thereby explaining the observed increase in viscosity (Figure 3). This hypothesis is consistent with observations made under more diluted conditions (Appendix A, Table A1). Adding 1% sodium chloride (NaCl) to a 1% solution of N2 and N2’ greatly increases the viscosity (i.e., from approximately 5000 mPa·s in pure water to around 21,000 mPa·s after adding 1% NaCl for N2 and from 2900 to around 11,000 mPa·s for N2’, as measured using a Brookfield LV Viscometer with Spindle 3 at Speed 6; New York, NY, USA). At a 2% concentration, the viscosity of N2 increased from 11,000 to 62,000 mPa·s with 1% NaCl, and N2’ rose from 13,000 to 40,000 mPa·s (Spindle 4, Speed 6). While not directly proven by these data, the speculative explanation was based on the observation that N2 consistently showed higher viscosity in the presence of salt at both tested concentrations and was further supported by the fact that the difference in viscosity between N2 and N2’ became more pronounced with increasing test dosage. Consequently, the rinsing procedure employing PBS may induce a localized elevation in viscosity, thereby leading to the retention of the polymer on the epidermal surface instead of its complete removal. This effect would have been notably more evident for N2 compared to N2’.

Besides laboratory testing, considering the current applications, xanthan gum is widely used in cosmetics. Indeed, it is the most commonly used natural polymer in cosmetic care formulations, with 3470 reported uses in 2016 compared to less than 347 reported uses for other polysaccharide gums [51]. Moreover, a more recent analysis using a formulation database indicates that xanthan gum was used in 41% of face and neck care formulations, 26.5% of body care formulations, and 22% of shampoos launched on the global market in the last five years [69]. Considering the widespread use of xanthan gum in skin care and personal care, cosmetovigilance reports are limited to one case of publicly reported contact allergy [70]. Based on the available information, the detected effect on N2 is likely due to increased epidermal contact caused by its sticky texture and rinsing difficulties, as visually observed by the technician during the test. Adjustments to the testing protocol may be considered to mitigate this effect, such as evaluating olive oil as a solvent. Furthermore, an additional preliminary assessment of texture could be implemented during the solvent selection process. In this preliminary stage, setting acceptability thresholds combined with a quantitative measurement would be optimal.

In general, one lesson from this polymer-specific work was that the polymer-testing format was important. Indeed, depending on its nature and manufacturing process, the behavior of the polymer when applied to the epidermis can be influenced by the dose and the choice of dilution solvent.

**Figure 4:**
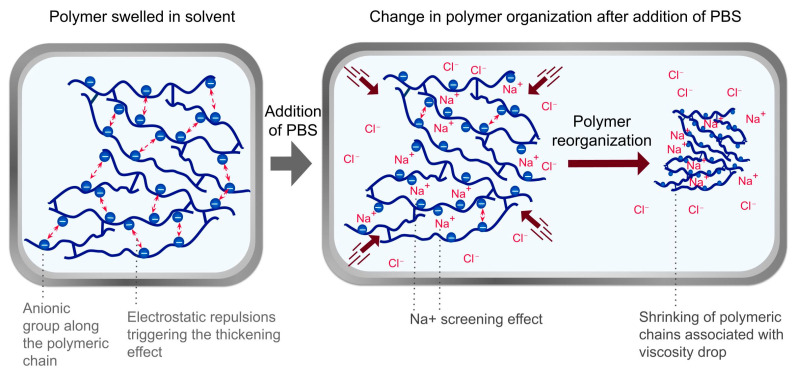
Salt screening effect in inverse emulsion polymer networks: hypothesis explaining the decreased viscosity and easy rinsing of preparations with polymers C1, C2, C3, and C4 during the Sens-Is assay.

On the one hand, as previously stated, the choice of solvent is important, as it affects the polymer’s state and its application on the epidermis. On the other hand, the efficiency of the PBS-washing step may vary depending on the sensitivity of the polymers to salts. Polymers C1, C2, C3, C4, and C5 were strongly affected by electrolytes (Appendix A, Table A1). Due to the presence of salts, PBS induces a decrease in viscosity that facilitates rinsing [71]. This phenomenon can be explained by the screening effect of Na+, which decreases the electrostatic repulsion between anionic charged groups located along the polymer chains, necessary for the thickening effect of these polymers (Figure 4).

Conversely, polymers N1, N3, N4, N5, R1, R2 (Appendix A, Table A1), and R3 [72] are not affected by the presence of a monovalent salt (i.e., the viscosity remains constant). As previously discussed, the presence of salts in PBS may increase the viscosity and cause difficulty in rinsing N2 and N2’ from the surface and epidermis, suggesting a point of vigilance. This may result in overexposure of the epidermis to the substance tested and may lead to an inconclusive response. The protocol modifications proposed in the preceding paragraph should be evaluated to determine if this limitation could be overcome. If not, an alternative test addressing the same key events may be considered.

These findings highlight the importance of including this first assessment as part of a multi-test approach, enabling the modulation and compensation for the specific limitations associated with each technique.

## 5. Conclusions

Both synthetic and natural rheology-modifiers were tested using the Sens-Is assay as a starting point for skin sensitization hazard assessment. Of the 15 polymers tested, only one triggered a positive response, while that polymer was classified as non-sensitizing in vivo. This discrepancy was attributed to technical difficulty in rinsing the tested preparation from the reconstructed epidermis model. Moreover, in the context of developing an experimental rheology modifier, the Sens-Is assay also demonstrated its capacity to detect impurities or secondary components in polymers that could potentially pose a risk of skin sensitization in humans. It revealed the method as a useful tool to optimize the final polymer composition. These findings indicate that the Sens-Is method is applicable for evaluating this family of rheology modifiers, allowing its inclusion in a multi-test strategy for assessing the skin sensitization hazard of novel rheology-modifying agents, such as modified natural gums.

This work also highlighted the importance of selecting the appropriate solubilising and rinsing solvents based on the polymer’s sensitivity to electrolytes. This criterion should be integrated in the preliminary step to avoid an inconclusive response. In the near future, the dataset can be extended to include other natural thickeners or rheology modifiers such as gellan, pullulan, pectin, locust bean gum, and modified cellulose to validate the efficacy of this remedial action, or the necessity of employing an alternative testing methodology should these rinsing challenges arise.

Additional tests addressing the remaining critical Key Events of the Adverse Outcome Pathway should be conducted on the same family of polymeric compounds to evaluate the predictive capacity of the strategy. This step is crucial in supporting the development of safe and high-performing polymers.

Furthermore, developing a model that more closely simulates in vivo skin application and aligns with the physiological characteristics of the organ would be a promising avenue for future research. Such a model could enhance the robustness of the biological system and the prediction of sensitizing effects.

## Figures and Tables

**Table 1:** Profile of the tested materials.

Polymer Identification	Polymer IUPAC Name	Polymerization Excipient (INCI Name)	Production Method	Physical Form	Polymer Content (*w*/*w* %)
C1 (synthetic)	1-Propanesulfonic acid, 2-methyl-2-[(1-oxo-2-propenyl)amino]-, monosodium salt, polymer with 2-propenamide	Inverse emulsion: -Water - Oil: Renewable Alkane - Inverter I1 *: Polyglyceryl-10 Laurate	Polymerization in inverse emulsion	Liquid	35–40
C2 (synthetic)	1-Propanesulfonic acid, 2-methyl-2-[(1-oxo-2-propenyl)amino]-, monosodium salt, polymer with 2-propenamide	Inverse emulsion: -Water - Oil: Isoparaffin - Inverter: Laureth-7	Polymerization in inverse emulsion	Liquid	35–40
C3 Experimental (synthetic)	2-Propenoic acid, polymer with 2-methyl-2-[(1-oxo-2-propenyl)amino]-1-propanesulfonic acid, sodium salt	Inverse emulsion: -Water - Oil: Renewable alkane - Inverter I2 *: Polyglyceryl-10 laurate	Polymerization in inverse emulsion	Liquid	35–40
C4 (synthetic)	2-Propenoic acid, polymer with 2-methyl-2-[(1-oxo-2-propenyl)amino]-1-propanesulfonic acid, sodium salt	Inverse emulsion: -Water - Oil: Renewable alkane - Inverter: Polyglyceryl-6 laurate	Polymerization in inverse emulsion	Liquid	35–40
C5 (synthetic)	2-Propenoic acid, polymer with 2-methyl-2-[(1-oxo-2-propenyl)amino]-1-propanesulfonic acid monosodium salt, 2-propenamide and sodium 2-propenoate	Inverse emulsion: -Water - Oil: Hydrogenated Polyisobutene and Ethylhexyl Palmitate - Inverter I1 *: Polyglyceryl-10 Laurate	Polymerization in inverse emulsion	Liquid	50–70
N1 (natural)	(2R,3R,4S,5R,6S)-2-(hydroxymethyl)-6-[[(2R,3S,4R,5S,6R)-4,5,6-trihydroxy-3-[(2S,3S,4S,5S,6R)-3,4,5-trihydroxy-6-(hydroxymethyl)oxan-2-yl]oxyoxan-2-yl]methoxy]oxane-3,4,5-triol	-	Extraction from seed of *Caesalpinia* *spinosa* tree	Powder	100
N2 ** (natural)	Not available INCI name: Xanthan gum	-	Biotechnology: fermentation of *Xanthomonas* *Campestris*	Powder	100
N2’ ** (natural)	Not available INCI name: Xanthan gum	-	Biotechnology: fermentation of *Xanthomonas* *Campestris*	Powder	100
N3 (natural)	(2R,3R,4S,5S,6R)-2-{[(2R,3S,4R,5R,6S)-6-{[(2R,3S,4R,5R,6R)-4,5-dihydroxy-6-{[(2R,3S,4R,5R,6S)-4,5,6-trihydroxy-2-(hydroxymethyl)oxan-3-yl]oxy}-3-{[(2R,3R,4S,5S,6R)-3,4,5-trihydroxy-6-(hydroxymethyl)oxan-2-yl]oxy}oxan-2-yl]methoxy}-4,5-dihydroxy-2-(hydroxymethyl)oxan-3-yl]oxy}-6-(hydroxymethyl)oxane-3,4,5-triol	-	Extraction from babassu mesocarp	Powder	100
N4 (natural)	(2S,3S,4S,5S,6R)-2-[(2R,3S,4R,5R,6S)-6-[(2R,3S,4R,5S,6S)-4,5-dihydroxy-2-(hydroxymethyl)-6-[(2R,4R,5S,6R)-4,5,6-trihydroxy-2-(hydroxymethyl)oxan-3-yl]oxyoxan-3-yl]oxy-4,5-dihydroxy-2-(hydroxymethyl)oxan-3-yl]oxy-6-(hydroxymethyl)oxane-3,4,5-triol	-	Extraction from roots of *Amorphophallus konjac*	Powder	100
N5 (natural)	(2S,3S,4S,5S,6R)-2-[(2R,3S,4R,5R,6S)-6-[(2R,3S,4R,5S,6S)-4,5-dihydroxy-2-(hydroxymethyl)-6-[(2R,4R,5S,6R)-4,5,6-trihydroxy-2-(hydroxymethyl)oxan-3-yl]oxyoxan-3-yl]oxy-4,5-dihydroxy-2-(hydroxymethyl)oxan-3-yl]oxy-6-(hydroxymethyl)oxane-3,4,5-triol	-	Extraction from roots of *Amorphophallus muelleri*	Powder	100
N6 (natural)	1,6,10-Dodecatriene, 7,11-dimethyl-3-methylene-, (6E)-, homopolymer, hydrogenated	Oil: Renewable alkane	Chemical transformation of sugar plant	Liquid	30–50
R1 (natural)	R1 (natural) [(2R,3R,4R,5R,6S)-4,5-dihydroxy-6-[[(1R,3R,4R,5S,8S)-3-[(2R,3S,4R,5R,6S)-5-hydroxy-2-(hydroxymethyl)-6-[[(1R,3S,4R,5S,8S)-3-hydroxy-4-sulfonatooxy-2,6-dioxabicyclo[3.2.1]octan-8-yl]oxy]-3-sulfonatooxyoxan-4-yl]oxy-4-sulfonatooxy-2,6-dioxabicyclo[3.2.1]oct	-	Extraction from red seaweeds	Powder	100
R2 (natural)	Not available (branched β-(1→3),(1→6)-D-glucan)	-	Biotechnology: fermentation of *Sclerotium rolfsii*	Powder	100
R3 (natural)	(2R,3S,4S,5R)-2-(hydroxymethyl)-6-[[(4R,5S)-4-hydroxy-3-methyl-2,6-dioxabicyclo[3.2.1]octan-8-yl]oxy]-4-methoxyoxane-3,5-diol	-	Extraction from red seaweeds	Powder	100
PR (synthetic)	2-ethylhexyl prop-2-enoate	-	-	Powder	100

* I1 and I2 are described by the same INCI name but obtained from different suppliers (I1 was obtained from Nikko Chemicals Co., Ltd., Tokyo, Japan; For ethical reasons related to the results, the supplier of I2 is not disclosed). ** N2 and N2’ are described by the same INCI name obtained from different suppliers.

**Table 2:** Results of Sens-Is assays.

	Conditions of Test		
Polymer	Solvents	Concentrations	Results
C1	PBS, DMSO, DPG	100%; 50%	Non sensitizer
C2	DMSO	100%; 50%	Non sensitizer
C3	DMSO	100%; 50%; 10%	Very weak sensitizer
C4	DMSO and PBS	100%; 50%; 10%	Non sensitizer
C5	DMSO	100%; 50%; 10%	Non sensitizer
N1	DMSO and PBS	100%	Non sensitizer
N2	DMSO and PBS	100%; 50%	Very weak sensitizer
N2’	DMSO	100%; 50%	Non sensitizer
N3	DMSO	100%; 50%; 10%	Non sensitizer
N4	DMSO	100%; 50%; 10%	Non sensitizer
N5	DMSO	100%; 50%; 10%	Non sensitizer
N6	Olive oil	100%; 50%; 10%	Non sensitizer
R1	DMSO and PBS	100%	Non sensitizer
R2	DMSO and PBS	100%	Non sensitizer
R3	DMSO and PBS	100%	Non sensitizer
PR	Olive oil, DPG	50%; 10%	Moderate sensitizer

**Table 3:** Comparison of Sens-Is and in vivo data available on tested items.

Polymer	Sens-Is Assay Results	Skin Sensitization Conclusion (Safety Analysis and In Vivo Results *)
C1	-	- ^a, c^ [47]
C2	-	- ^a, c^ [48]
C3	Very weak sensitizer	Not expected
C4	-	- ^c^ [49]
C5	-	- ^a, b, c^ [50]
N1	-	- ^b^
N2	Very weak sensitizer	- ^a^ [51]
N2’	-	- ^a^ [51]
N3	-	- ^c^ [52]
N4	-	- ^a^ [34]
N5	-	- ^c^ [53]
N6	-	- ^c^ [52]
R1	-	- ^a^ [34]
R2	-	- ^a^ [33]
R2	-	- ^a^ [34]
PR	Moderate sensitizer	Moderate sensitizer ^b^ [28]

-: Non-sensitizer, ^a^: [54], ^b^: [55], ^c^ [47,48,49,50,52]. *: The guideline followed for each test is indicated by a, b, or c. Published data for the substances are indicated by bracketed references [x] within the table. Data without these notes are based on internal findings.

## Data Availability

Data are contained within the article. The raw data supporting the conclusions of this article will be made available by the authors upon request.

## Abbreviations

The following abbreviations are used in this manuscript:

3D	Three Dimensional
ACD	Allergic Contact Dermatitis
AOP	Adverse Outcome Pathway
ARE	Antioxidant/electrophile Response
C1 to C6	Synthetic polymers
Ca2+	Calcium cation
CaCl2	Calcium Chloride
cDNA	complementary Deoxyribonucleic Acid
Cl^−^	Chlorine anion
Da	Dalton
DMSO	DiMethylvitro SulfOxide
DPG	Dipropylene Glycol
ECHA	European Chemicals Agency
HRIPT	Human Repeated Insult Patch Test
INCI	International Nomenclature Cosmetic Ingredient
KE	Key Events
N1 to N6	Natural polymers (Composition in Table 1)
Na+	Sodium cation
NaCl	Sodium Chloride
NMR	Nuclear Magnetic Resonance
OECD	Organisation for Economic Co-operation and Development
PBS	Phosphate Buffered Saline
PR	Positive Reference
R1 to R3	References of natural polymers (Composition in Table 1)
RT-PCR	Reverse Transcriptase—Polymerase Chain Reaction
SOP	Standard Operating Protocol
TNBS	2,4,6-trinitrobenzene sulfonic acid
UVCB	Unknown or Variable composition, Complex reaction products or Biological materials

## Appendix A

The following Table A1 summarizes the influence of salt addition on the viscosity of diluted hydrogels. Thess data have informed the speculations presented in the discussion.

**Table A1:** Influence of salt on the viscosity of aqueous gels (pH 5–6.5).

Polymer	Dose	Viscosity * in Demineralized Water (in mPa·s)	Viscosity * in Demineralized Water + 0.6% NaCl (in mPa·s)	Viscosity * in Demineralized Water + 1% NaCl (in mPa·s)	Trend **: Viscosity Evolution After NaCl Addition
C1	2%	79,000	<50	<50	➘ Decrease
C2	2%	155,000	10,000	4500	➘ Decrease
C3	2%	90,000	<50	<50	➘ Decrease
C4	2%	52,000	<50	<50	➘ Decrease
C5	2%	90,000	500	<50	➘ Decrease
N1	1%	4700	Not done	4500	➞ Constant
N2	1%	5000	Not done	21,000	➚ Increase +
	2%	11,000	Not done	62,000	➚ Increase +
N2’	1%	2900	Not done	11,000	➚ Increase
	2%	13,000	Not done	40,000	➚ Increase
N3	1%	<50	Not done	<50	➞ Constant
N4	1%	24,000	Not done	17,000	➞ Constant
N5	1%	44,000	Not done	41,000	➞ Constant
N6	1%	Oil rheology modifier—Not soluble in water, no effect of salts			
R1	1%	14,500	Not done	18,000	➞ Constant
R2	1%	8000	Not done	8500	➞ Constant

* Brookfield LV, with Spindle 2 at Speed 6 for viscosities less than 5000 mPa·s; with Spindle 3 at Speed 6 for viscosities between 5000 mPa·s and 20,000 mPa·s; with Spindle 4 at Speed 6 for viscosities between 10,000 mPa·s and 100,000 mPa·s; Brookfield RV, Spindle 7 at Speed 5 for viscosities greater than 100,000 mPa·s. ** A 20% error margin in the Brookfield viscosities was considered when determining the trend.
